# Software Carpentry: lessons learned

**DOI:** 10.12688/f1000research.3-62.v2

**Published:** 2016-01-28

**Authors:** Greg Wilson

**Affiliations:** 1 Software Carpentry Foundation, Austin, TX, USA

**Keywords:** Software Carpentry, Scientific Computing, Training, Education

## Abstract

Since its start in 1998, Software Carpentry has evolved from a week-long training course at the US national laboratories into a worldwide volunteer effort to improve researchers' computing skills. This paper explains what we have learned along the way, the challenges we now face, and our plans for the future.

## 1 Introduction

In January 2012, John Cook posted this to his widely-read blog ^1^:

In a review of linear programming solvers from 1987 to 2002, Bob Bixby says that solvers benefited as much from algorithm improvements as from Moore’s law: “Three orders of magnitude in machine speed and three orders of magnitude in algorithmic speed add up to six orders of magnitude in solving power. A model that might have taken a year to solve 10 years ago can now solve in less than 30 seconds.”

A million-fold speed-up is impressive, but hardware and algorithms are only two sides of the iron triangle of programming. The third is programming itself, and while improvements to languages, tools, and practices have undoubtedly made software developers more productive since 1987, the speed-up is percentages rather than orders of magnitude. Setting aside the minority who do high-performance computing (HPC), the time it takes the “desktop majority” of scientists to produce a new result is frequently dominated by how long it takes to write, test, debug, install, and maintain software.

The problem is that most scientists are never taught how to do this. Their undergraduate programs may include a generic introduction to programming or a statistics or numerical methods course (in which they are often expected to pick up programming on their own), but they are almost never told that version control exists, and rarely if ever shown how to structure a maintainable program, or how to turn the last twenty commands they typed into a re-usable script. As a result, they routinely spend hours doing things that could be done in minutes, or don’t do things at all because they don’t know where to start ^2, 3^.

This is where Software Carpentry comes in. We ran over 400 workshops for over 12,000 researchers between January 2013 and July 2015. In them, over 400 volunteer instructors helped attendees learn about program design, task automation, version control, and other unglamorous but time-tested skills ^4^. Two independent assessments in 2012 ^5, 6^ and two others more recently ^7, 8^ have indicated that this training is helping (though as we discuss in Section 8.1, these results are still preliminary):

The program increases participants’ computational understanding, as measured by more than a two-fold (130%) improvement in test scores after the workshop. The program also enhances their habits and routines, and leads them to adopt tools and techniques that are considered standard practice in the software industry. As a result, participants express extremely high levels of satisfaction with their involvement in Software Carpentry (85% learned what they hoped to learn; 95% would recommend the workshop to others).

## 2 From red to green

Like many projects, it has taken us years to become an overnight success, and we have made many mistakes along the way. These are best understood historically.

### 2.1 Version 1: red light

In 1995–96, the author organized a series of articles in *IEEE Computational Science & Engineering* titled, “What Should Computer Scientists Teach to Physical Scientists and Engineers?” ^9^. These grew out of the frustration he had working with scientists who wanted to run before they could walk, i.e., to parallelize complex programs that were not broken down into self-contained functions, that did not have any automated tests, and that were not under version control ^10^.

In response, John Reynders (then director of the Advanced Computing Laboratory at Los Alamos National Laboratory) invited the author and Brent Gorda (now at Intel) to teach a week-long course to LANL staff. This course ran for the first time in July 1998, and was repeated nine times over the next four years. It eventually wound down as Gorda and the author moved on to other projects, but two valuable lessons were learned:

1. Intensive week-long courses are easy to schedule (particularly if instructors have to travel) but by the last two days, attendees’ brains are full and learning drops off significantly.
2. Textbook software engineering is not useful to most scientists. In particular, careful documentation of requirements and lots of up-front design are not appropriate for people who (almost by definition) do not know what the right answer is yet. Agile development methods (which rose to prominence during this period) are a less bad fit to researchers’ needs, but even they are not well suited to the common “solo grad student” model of working.

### 2.2 Versions 2 and 3: Another red light

The Software Carpentry course materials were updated and released in 2004–05 under a Creative Commons license with support from the Python Software Foundation ^11^. They were used twice in a conventional term-long graduate course at the University of Toronto aimed at a mix of students from Computer Science and the physical and life sciences.

The materials attracted 1000–2000 unique visitors a month. But while graduate students (and the occasional faculty member) found the course at Toronto useful, it never found an institutional home. Most Computer Science faculty believe that this basic material is too easy to deserve a graduate credit (even though a significant minority of their students, particularly those coming from non-CS backgrounds, have no better software development skills than the average physicist). Meanwhile, other departments believe that courses like this ought to be offered by Computer Science, in the same way that Mathematics and Statistics departments routinely offer service courses. In the absence of an institutional mechanism to offer credit courses at some inter-departmental level, this course, like many other interdisciplinary initiatives, was left without a home.

**It works too well to be worth teaching**
  Most computer scientists want to do research to advance our understanding of the science of computing; things like command-line history, tab completion, and “select * from table” have been around too long, and work too well, to be interesting. As long as universities reward research first, and teaching last, it is simply not in most computer scientists’ interests to offer courses like this.

Secondly, despite repeated invitations, other people did not contribute new material beyond an occasional bug report (a point which we will return to in Section 6).

The most important lesson, though, was that while many faculty in science, engineering, and medicine agree that their students should learn more about computing, they *won’t* agree on what to take out of the current curriculum to make room for it. A typical undergraduate science degree in the US or Canada comprises roughly 1800 hours of class and laboratory time. Anyone who wants to add more programming, statistics, writing, or anything else must either lengthen the program (which is financially and institutionally infeasible) or take something out. However, everything in the program is there because it has a passionate defender who thinks it’s vitally important, and who is likely senior to those faculty advocating the change.

**It adds up**
Saying, “We’ll just add a little computing to every other course,” is a cheat: five minutes per hour equals four entire courses in a four-year program, which is unlikely to ever be implemented. Pushing computing down to the high school level is also a non-starter, since that curriculum is also full.

The sweet spot for this kind of training is therefore the first years of graduate school. At that point, students have time to learn (at least, more time than they’ll have once they’re faculty) and real problems of their own that they want to solve.

### 2.3 Version 4: orange light

The author rebooted Software Carpentry in May 2010 with support from Indiana University, Michigan State University, Microsoft, MITACS, Queen Mary University of London, Scimatic, SciNet, SHARCNet, and the UK Met Office. More than 120 short video lessons were recorded during the subsequent 12 months, and six week-long classes were run for the backers. We also offered an online class three times (a MOOC *avant la lettre*).

This was our most successful version to date, in part because the scientific landscape itself had changed. Open access publishing, crowd sourcing, the data deluge in the life sciences, and growing concern about reproducible research had convinced a growing number of scientists that knowing how to program was now as important as knowing how to do statistics. Even most of them, though, still (rightly) regarded it as a tax they had to pay in order to get their science done.

Despite this round’s overall success, there were several disappointments:

1. Once again, we discovered that five eight-hour days are more wearying than enlightening.
2. And once again, only a handful of other people contributed material (see Section 6).
3. Creating videos is significantly more work than creating slides. Editing or modifying them is harder still: while a typo in a slide can be fixed by opening PowerPoint, making the change, saving, and re-exporting the PDF, inserting new slides into a video and updating the soundtrack seems to take at least half an hour regardless of how small the change is. This makes maintaining a video-based course prohibitively expensive.
4. Most importantly, the MOOC format didn’t work: only 5–10% of those who started with us completed the course, and the majority were people who already knew most of the material. Both figures are in line with completion rates and learner demographics for other MOOCs ^12^, but that does not make them less disappointing.

The biggest take-away from this round was the need to come up with a scalable, sustainable model for delivering training. One instructor simply can’t reach enough people, and cobbling together funding from half a dozen different sources every twelve to eighteen months is risky as well as wearying.

### 2.4 Version 5: green light

Software Carpentry rebooted again in January 2012 with a grant from the Sloan Foundation to the Mozilla Foundation. This time, the model was two-day intensive workshops like those pioneered by The Hacker Within, a grassroots group of grad students helping grad students at the University of Wisconsin - Madison.

Shortening the workshops made it possible for more people to attend, and increased the proportion of the material they could absorb. It also forced us to think much harder about what skills scientists really needed. Out went object-oriented programming, XML, Make, and other topics. Instead, we focused on a small set of tools that let us introduce higher-level concepts without learners really noticing ( Section 3).

Reaching more people allowed us to recruit new instructors from workshop participants, which in turn allowed us to scale. Switching to a “host site covers costs” model was equally important: funding was still needed for 1.5 core staff to lead the project and match instructors to workshops, but everything else funded itself.

**Learning to teach**
One of our most important discoveries during this period was that many people are as interested in learning about better teaching practices as they are in learning about computing. We discuss this in detail in Section 5.

### 2.5 Version 6: A true community project

In July 2014, the author left Mozilla and set up the Software Carpentry Foundation, an independent non-profit foundation under the auspices of NumFOCUS. The SCF held its first elections in January 2015, in which instructors who had taught over the past two years selected seven of their own number as a Steering Committee to oversee the project’s operations. Since then, the SCF has formed partnerships with a growing number of institutions (see Table 1), run an ever-increasing number of workshops, and much more.

**Table 1.** Current Partners (November 2015).

Berkeley Institute for Data Science
Compute Canada
GitHub
Insight Data Science
iPlant
Lawrence Berkeley National Laboratory
Michigan State University
Netherlands eScience Center
New Zealand eScience Infrastructure
Oklahoma State University
RStudio
Software Sustainability Institute
University College London
UCAR
University of California Davis
University of Colorado
University of Florida
University of Leeds
University of Melbourne
University of Michigan
University of Oklahoma
University of Washington

While the SCF is only nine months old, we have already learned many things. The most important are:

1. The first few people to join a volunteer organization are usually keener than those who join later. As numbers grow, therefore, the time contributed per person will decrease, and structures must be designed with this in mind. In particular, by the time 400 people are involved, most will be dipping in and out of conversations rather than taking part on a daily or weekly basis, so frameworks and procedures must become simple and stable.
2. Every partner organization has different needs and constraints (We have learned much more than we ever wanted to about accounting rules at various universities. . .). “Standard” partnership agreements therefore have to be treated as starting points for negotiation, rather than as “take it or leave it” propositions.
3. “Bikeshedding” is the practice of arguing over minor, marginal issues while more serious ones are overlooked. It is a constant danger in an organization whose more vocal members actually enjoy programming. Squelching such technical discussions has a chilling effect on conversation overall, but letting them go unchecked alienates people who would rather talk about teaching, or simply don’t have enough time to go down technical rabbit holes. We discuss an example in Section 7 and Section 8.4.

### 2.6 Data Carpentry

The biggest recent development, though, has been the foundation of a sibling organization called Data Carpentry in April 2014. Where Software Carpentry’s mission is to help scientists who are programming badly to program better, Data Carpentry’s focus is, as its name implies, to help them manage and analyze their data. Led by Dr. Tracy Teal, Data Carpentry was recently awarded $700,000 by the Moore Foundation, and is expected to grow rapidly over the coming two years.

### 2.7 Results

Cumulative Number of Workshops over Time The cumulative number of Software Carpentry workshops between November 2011 – October 2015, and the dates they were held. Click here for additional data file. Copyright: © 2016 Wilson G 2016 Data associated with the article are available under the terms of the Creative Commons Zero "No rights reserved" data waiver (CC0 1.0 Public domain dedication).

Cumulative Number of Workshop Attendees over Time The cumulative number of people attending Software Carpentry workshops between November 2011 – October 2015. Click here for additional data file. Copyright: © 2016 Wilson G 2016 Data associated with the article are available under the terms of the Creative Commons Zero "No rights reserved" data waiver (CC0 1.0 Public domain dedication).

Cumulative Number of Qualified Instructors over Time The cumulative number of qualified instructors trained between May 2012 – October 2015. Click here for additional data file. Copyright: © 2016 Wilson G 2016 Data associated with the article are available under the terms of the Creative Commons Zero "No rights reserved" data waiver (CC0 1.0 Public domain dedication).

**Figure 1.**
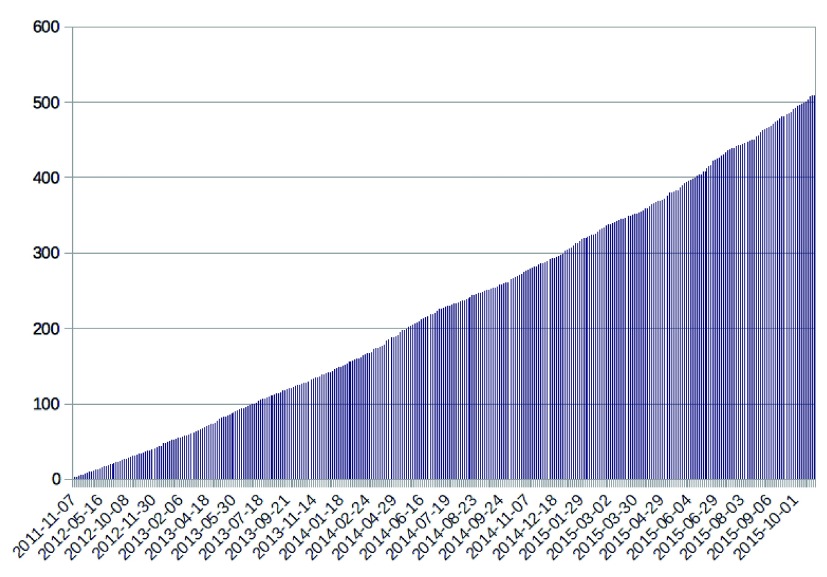
Cumulative number of workshops.

**Figure 2.**
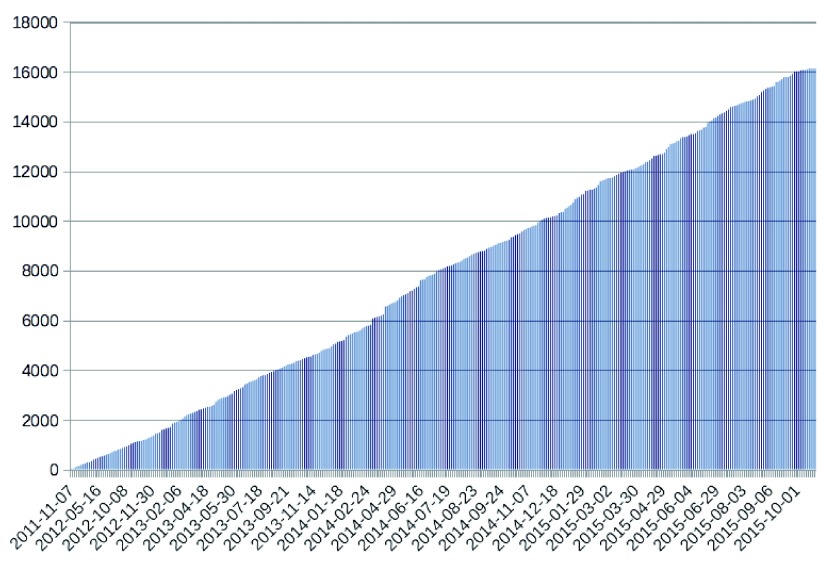
Cumulative number of learners.

**Figure 3.**
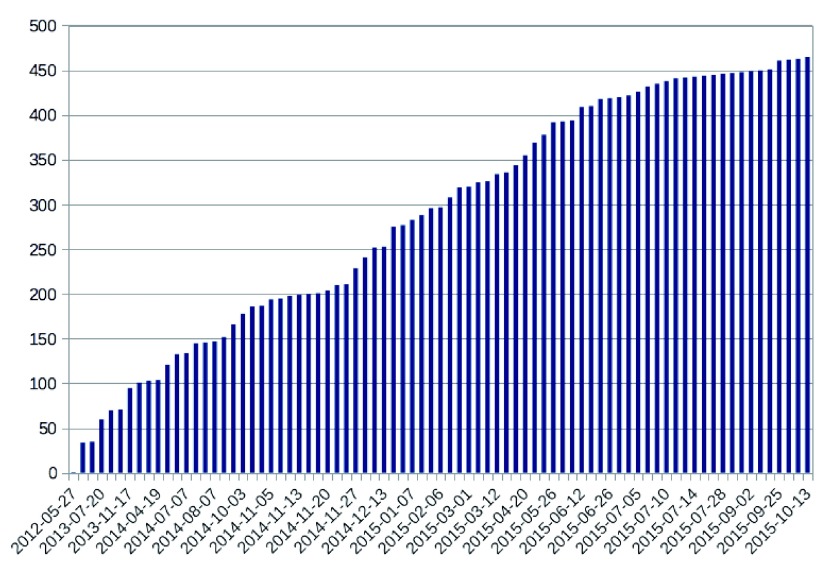
Cumulative number of instructors.

As we discuss in Section 8.1, we do not know how to measure the impact of our workshops. However, both their number ( Figure 1), and the number of people attending ( Figure 2), have grown steadily, as has the number of instructors ( Figure 3).

We are now a truly global organization ( Table 3). And most importantly, feedback from participants is strongly positive. While there are always problems with software set-up and the speed of instruction ( Section 8.2), 80–90% of attendees typically report that they were glad they attended and would recommend the workshops to colleagues.

**Table 2.** Authors per Lesson (October 2015).

Topic	Contributors
Git	55
Mercurial	25
MATLAB	28
Python	52
R	49
Unix Shell	64
SQL	41

**Table 3.** Workshops and Instructors by Country (October 2015).

Country	Workshops	Instructors
United States	216	232
Canada	59	52
United Kingdom	43	50
Australia	33	41
Brazil	9	2
South Africa	6	1
New Zealand	5	8
Norway	5	3
Germany	5	9
South Korea	4	1
France	3	3
Poland	3	5
Switzerland	3	0
Italy	2	1
Netherlands	2	0
Spain	2	3
China	1	1
Cyprus	1	0
Denmark	1	2
Finland	1	0
Ghana	1	0
Indonesia	1	0
Jordan	1	0
Lebanon	1	0
Saudi Arabia	1	0
Sweden	1	2
Thailand	1	2
India	0	1

## 3 What we do

So what does a typical workshop look like?

- *Day 1 a.m.*: The Unix shell. We only show participants a dozen basic commands; the real aim is to introduce them to the idea of combining single purpose tools (via pipes and filters) to achieve desired effects, and to getting the computer to repeat things (via command completion, history, and loops) so that people don’t have to.
- *Day 1 p.m.*: Programming in Python, R, or MATLAB. (Only one language is taught in any given workshop.) The real goal is to show them when, why, and how to grow programs step-by-step as a set of comprehensible, reusable, and testable functions.
- *Day 2 a.m.*: Version control. We begin by emphasizing how this is a better way to back up files than creating directories with names like “final”, “really_final”, “really_final_revised”, and so on, then show them that it’s also a better way to collaborate than FTP or Dropbox.
- *Day 2 p.m.*: Either more about programming in the workshop’s chosen language, or an introduction to databases and SQL. If the latter is chosen, the real goal is to show them what structured data actually is (in particular, why atomic values and keys are important) so that they will understand why it’s important to store information this way.

As the descriptions above suggest, our real aim isn’t to teach any specific tool: it’s to teach *computational competence*. We can’t do this in the abstract: people won’t show up for a hand-waving talk about general principles because they won’t believe those principles will help them meet next Thursday’s deadline. Even if they do, they won’t understand, because big ideas need to be grounded in specific examples to be comprehensible. If we show them how to solve a specific problem with a specific tool, we can then lead into a larger discussion of how scientists ought to develop, use, and curate software.

There are a lot of local variations around the curriculum shown above. For example, some instructors use the command-line Python interpreter, while others prefer the Jupyter Notebook. Still others teach R or MATLAB instead, while a handful of workshops also cover tools such as LaTeX, or domain-specific topics such as audio file processing, depending on the needs of the audience and the expertise of the instructor.

We aim for no more than 40 people per room at a workshop, so that every learner can receive personal attention when needed. Where possible, we run two or more rooms side by side, and use a pre-assessment questionnaire to stream learners by prior experience, which simplifies teaching and improves their experience. We do *not* shuffle people from one room to another between the first and second day: with the best inter-instructor coordination in the world, doing so would result in lost context.

Our workshops are sometimes free, but most now charge a small registration fee (typically $20–40), primarily because it reduces the no-show rate from a third to roughly 5%. When this is done, we must be careful not to trip over institutional rules about commercial use of their space: some universities will charge hundreds or thousands of dollars per day for use of their classrooms if any money changes hands. As this is usually several times more than a small registration fee would bring in, we usually choose the higher no-show rate as the lesser evil (We have also experimented with refundable deposits, but the administrative overheads were unsustainable. It also does not help get around the rules mentioned in the previous paragraph, since money still appears to be changing hands in the university’s eyes.).

**Commercial offerings**
Our material ^13, 14^ is all covered by the Creative Commons Attribution license, so anyone who wants to use it for commercial training can do so without explicit permission from us. We encourage this: if graduate students can help pay their bills by sharing what they know, in the way that many programmers earn their living by working on open source software, our community will only be stronger.
What *does* require permission is use of our name and logo, both of which are trademarked. Such permission is granted automatically if at least one instructor is certified, the workshop covers three core topics (the shell, version control, and a programming language), and the organizers send us summary information (the dates, the location, and the number of attendees). We put these rules in place because of people calling something “Software Carpentry” when they had nothing to do with what we usually teach. We have worked hard to create material that actually helps scientists, and to build some name recognition around it, and we would like to make sure our name continues to mean something.
**Administration fees**
If the Software Carpentry Foundation helps to organize a workshop (e.g., finds instructors and handles registration) then we charge the host site a $2500 administration fee. This fee, which currently provides about a quarter of our revenue, is routinely waived for workshops in under-served areas and developing countries. If host sites organize the workshop themselves, we will still set up registration and send out pre and post-workshop questionnaires. There is no fee in this case, but we do ask for a donation (we suggest $500).

As well as instructors, we rely on local helpers to wander the room and answer questions during practical sessions. These helpers may be alumni of previous workshops who are interested in becoming instructors, grad students who have picked up some or all of our core skills on their own, or members of the local open source community; where possible, we aim to have at least one helper for every eight learners.

We find workshops go a lot better if people come in groups (e.g., 4–5 people from one lab) or have other pre-existing ties (e.g., are working in the same field). They are less inhibited about asking questions, and can support each other (morally and technically) when the time comes to put what they’ve learned into practice after the workshop is over. Group sign-ups also yield much higher turnout from groups that are otherwise often under-represented, such as women and minority students, since they know in advance that they will be in a supportive environment.

## 4 Small things add up

As in chess, success in teaching often comes from the accumulation of seemingly small advantages. Here are a few of the things we do that we believe have contributed to our success.

### 4.1 Feedback loops

Giving each learner two sticky notes of different colors allows instructors to do quick true/false questions as they’re teaching. It also allows real-time feedback during hands-on work: learners can put a green sticky note on their laptop when they have something completed, or a red one when they need help.

We also use them as minute cards: before each break, learners take a minute to write one thing they’ve learned on the green sticky note, and one thing they found confusing (or too fast or too slow) on the red. It only takes a couple of minutes to collate these, and allows the instructors to adjust to learners’ interests and speed.

We frequently also ask for summary feedback at the end of each day. The instructors ask the learners to alternately give one positive and one negative point about the day, without repeating anything that has already been said. This requirement forces people to say things they otherwise might not: once all the “safe” feedback has been given, participants will start saying what they *really* think.

**Different channels, different messages**
Minute cards are anonymous; the alternating up-and-down feedback is not. Each mode has its strengths and weaknesses, and by providing both, we hope to get the best of both worlds.

On a longer timescale, we send a post-workshop assessment questionnaire to attendees shortly after the workshop ends. Response rates vary, but are usually low, and the opt-in nature of the survey undoubtedly biases the data ( Section 8.1). Feedback from instructors has proven more insightful. In August 2015, for example, Azalee Bostroem surveyed our instructors to find out what they were actually teaching about Python ( http://software-carpentry.org/blog/2015/09/thinking-about-teaching.html). From this, we learned that 60% of our learners are novices with little or no prior programming experience, and that only a third of workshops get through the entire Python lesson.

Finally, starting in January 2015 we began running biweekly debriefing sessions for instructors who have recently taught workshops, in which they can discuss what they actually did, how it worked, how the lessons they actually delivered differed from our templates, what problems arose, and so on. Summaries are posted shortly after each meeting, and Alistair Walsh recently collected and posted information about the same Python lesson discussed above ( http://software-carpentry.org/blog/2015/10/python-debriefing-summary.html). We are now (October 2015) beginning a redesign of the lesson to take all this information into account.

### 4.2 Live coding

We teach via live coding rather than using slides because:

- Watching code emerge on the screen is much more convincing than looking at pre-made slides.
- It enables instructors to be more responsive to “what if?” questions.
- It facilitates lateral knowledge transfer (e.g., people learn about keyboard shortcuts and efficient search/replace strategies in the editor as well as Python).
- It slows instructors down: if they have to type in code as they go along, they can only go twice as fast as their learners instead of ten times as fast. (And once instructors get in the habit of saying everything twice—once as they’re typing, and a second time to recapitulate, pointing at the screen—most learners are able to keep up.)
- Learners get to see instructors’s mistakes *and how they diagnose and fix them*. Learners frequently report that this is the most valuable part of the workshop: as novices, they’re going to spend most of their time trying to figure out what’s gone wrong and how to fix it, so it’s very valuable to see which parts of error messages instructors pay attention to, and what steps they take to correct mistakes.

It takes a bit of practice for instructors to get used to thinking aloud while coding in front of an audience, but most report that it is then no more difficult to do than talking off a deck of slides.

**One device good, two devices better**
Many instructors now use two devices when teaching: a laptop plugged into the projector for learners to see, and a tablet beside it on which they can view their notes and the Etherpad session ( Section 4.7). This seems to be more reliable than displaying one virtual desktop while flipping back and forth to another.

### 4.3 Open everything

Our grant proposals, mailing lists, and everything else that isn’t personally sensitive are out in the open (see 13 for links). We believe that letting people see us succeed, fail, and learn encourages them to be more involved in our community, and inspires them to be open as well.

### 4.4 Open lessons

This is an important special case of the previous point. Anyone who wants to use our lessons can take what we have, make changes, and offer those back by sending us a pull request on GitHub. As discussed in Section 6, this workflow is foreign to most educators, but allows us to scale and adapt more quickly and more cheaply than the centralized approaches being taken by many high-profile online education ventures.

For example, we recently “published” our core lessons through Zenodo. The number of contributors per lesson is shown in Table 2. The distribution of contributions has the usual long-tail distribution, but the fact remains that our lessons have had more contributors than many “massive” and “open” online courses.

### 4.5 Use what we teach

We also make a point of eating our own cooking, e.g., we use GitHub for our web site and to plan workshops. Again, this makes us more credible, and gives instructors hands-on practice with the things they’re going to teach. Up until a year ago, the (considerable) downside to this was that it could be difficult for newcomers to contribute material. We have simplified our templates and build procedures considerably to fix this, and will be making more changes early in 2016 to incorporate further insights.

One problem we haven’t solved is the bikeshedding mentioned earlier. Many contributors would rather spend days tweaking the build process for lessons rather than an hour coming up with some new self-test exercises for those same lessons, both because they are on more familiar ground when debating programming issues, and because the feedback loop is much tighter. One of our goals for the coming year is to push the bulk of discussion toward teaching practices and lesson content.

### 4.6 Meet the learners on their own ground

Learners tell us that it is important to them to leave the workshop with their own machine set up to do real work. We therefore continue to teach on all three major platforms (Linux, Mac OS X, and Windows), even though it would be simpler to require learners to use just one ( Section 8.3).

We have experimented with virtual machines (VMs) on learners’ computers to reduce installation problems, but those introduce problems of their own: older or smaller machines simply aren’t fast enough, and learners often struggle to switch back and forth between two different sets of keyboard shortcuts for things like copying and pasting.

Some instructors use VPS over SSH or web browser pages instead. This solve the installation issues, but makes us dependent on host institutions’ WiFi (which can be of highly variable quality), and has the issues mentioned above with things like keyboard shortcuts.

### 4.7 Collaborative note-taking

We often use Etherpad for collaborative note-taking and to share snippets of code and small data files with learners. (If nothing else, it saves us from having to ask students to copy long URLs from the presenter’s screen to their computers.) It is almost always mentioned positively in post-workshop feedback, and several workshop participants have started using it in their own teaching.

### 4.8 Pair programming

Pairing is a good practice in real life, and an even better way to teach: partners can not only help each other out during the practical, but can also clarify each other’s misconceptions when the solution is presented, and discuss common research interests during breaks. To facilitate this, we strongly prefer flat (dinner-style) seating to banked (theater-style) seating; this also makes it easier for helpers to reach learners who need assistance.

### 4.9 Diversity

On June 24–25, 2013, we ran our first workshop for women in science, engineering, and medicine. This event attracted 120 learners, 9 instructors, a dozen helpers, and direct sponsorship from several companies, universities, and non-profit organizations. Our second such workshop ran in March 2014, and we have done half a dozen of varying sizes since. While we do occasionally get complaints (mostly from outsiders) about such events being discriminatory, they are overwhelmed by the uniformly positive response from participants, many of whom say that they would probably not have attended a mixed-gender event because of previous bad experiences with tech meetups.

## 5 Instructor training

The instructor training program that we started in August 2012 has attracted hundreds of participants, and at the time of writing there are over 400 more on the waiting list. This introduction to modern research in education and evidence-based teaching practices ^15^ doesn’t just improve our teaching: it also helps give the instructors a sense of community and purpose.

In its original form, training took 2–4 hours/week of participants’ time for 12–14 weeks (depending on scheduling interruptions); more recently, we have run it both as a live two-day event, and as a two-day online event, in which participants are together in groups of half a dozen or more at one, two, or three sites, while the instructor takes part over the web.

This training course introduces participants to the basics of educational psychology, instructional design, and how these things apply to teaching programming ^16– 20^. It is necessarily very shallow, but most participants find the material interesting as well as useful. Introducing grad students and faculty to evidence-based teaching practices may turn out to be Software Carpentry’s greatest contribution.

### 5.1 Why teach?

But why do people volunteer as instructors?

- *To make the world a better place.* The two things we need to get through the next hundred years are more science and more courage; by helping scientists do more in less time, we are helping with the former.
- *To make their own lives better.* Our instructors are often asked by their colleagues to help with computing problems. The more those colleagues know, the more interesting those requests are.
- *To network.* Showing up to run a workshop is a great way for people to introduce themselves to colleagues and make contact with potential collaborators. This is probably the most important reason from Software Carpentry’s point of view, since it’s what makes our model sustainable.
- *To practice teaching.* This is also important to people contemplating academic careers.
- *To help diversify the pipeline.* Computing is 12–15% female, and that figure has been *dropping* since its high point in the 1980s ^21^. Some of our instructors are involved in part because they want to help break that cycle by participating in activities like our workshops for women in science and engineering.
- *To learn new things, or learn old things in more detail.* Working alongside an instructor with more experience is a great way to learn more about the tools, as well as about teaching.
- *It’s fun.* Our instructors get to work with smart people who actually want to be in the room, and don’t have to mark anything afterwards. It’s a refreshing change from teaching undergraduate calculus. . .

## 6 Collaborative lesson development

Large-scale ad hoc collaboration is the norm in open source software development and the creation of encyclopedia articles, but is still rare in other fields. In particular, teachers often use one another’s slide decks as starting points for their own courses, but rarely offer their changes back to the original author in order to improve them. This is only partly because educators’ preferred file formats (Word, PowerPoint, and PDF) aren’t handled gracefully by existing version control systems. A deeper cause is that there isn’t a culture of contribution, particularly in higher education.

The question is, why not? Reasons advanced include:

- *Lack of technical skill.* But (a) many teachers edit Wikipedia, and (b) a large number of those who teach programming certainly *do* have the technical skills.
- *Lack of institutional rewards.* But if this was a real barrier, open source software and Wikipedia wouldn’t exist.
- *Episodic interaction.* If someone is teaching a full or half-year course, they may only revisit the material every six months to a year, and the context in which it’s taught may well be different.
- *It just hasn’t happened yet.* This argument might have been tenable a decade ago, but is less credible with every passing year.

Our current hypothesis is that teaching is *enacted knowledge* ^22, 23^. To make a musical analogy, the lesson plan, slides, and assignments are only the score; what matters most is how it’s performed. If this is correct, then collaborative lesson development will only succeed if it is done as part of what the Japanese call *jugyokenkyu* (lesson study): the systematic observation and discussion of lessons by fellow teachers.

In aid of this, in January 2015 we began running biweekly debriefing sessions for instructors who have recently taught workshops (see Section 4.1). We are also planning to revise instructor training to require trainees to watch and reflect on videos of experienced instructors delivering our lessons. We hope that making this “the new normal” will encourage even more collaboration on the content and delivery of our lessons.

## 7 Example: lesson templates

Section 2.5 mentioned that we have spent more time wrangling over technical details (“bikeshedding”) than we should have, at the expense of discussing pedagogy and lesson content. The prime example of this is probably the way we format our lessons: we have invested hundreds of hours in debating and implementing various options. Over the years, we have tried the following:

- *HTML.* People (rightly) complained about editing HTML tags was annoying, and about maintaining forward/backward links and glossary entries by hand.
- *XML with a custom translation tool.* This had all the disadvantages of HTML, with extra overhead of maintaining the XML-to-HTML translation tool.
- *A wiki.* The tool used didn’t handle concurrent edits gracefully, and didn’t provide any mechanism for prepublication review. We could live without the former if the latter worked, but the wiki tools available at the time also didn’t provide a way to indicate the semantics of specific regions, e.g., to signal that this part of the lesson was the objectives, while that was an exercise.

- *All lessons in one big repository.* This was unsatisfactory for (at least) three reasons:
  1. Putting everything in one repository made that repository uncomfortably large to clone.
  2. If people subscribed to notifications for the repository, they were inundated with notices about changes to lessons they didn’t care about.

At the same time, we experimented with using http://jupyter.org/ to author lessons. Notebooks are a wonderful tool for doing real scientific work, but less well suited to large-scale collaboration. In particular, while it’s possible for experienced users to diff and merge Jupyter Notebooks, it is intimidating and error-prone for newcomers (particularly in the face of embedded images) (The irony of telling people not to use “binary” formats like Microsoft Word for documents because they don’t play nicely with version control, and then using a format that is almost as awkward, did not escape our users. . .).

- *Markdown and HTML in a single GitHub repository per lesson with a custom build.* Markdown files in the gh-pages branch of a GitHub repository will be automatically translated into HTML using a tool called Jekyll, and those HTML pages will then be published as a website. This is great—except that Jekyll can’t translate Jupyter Notebooks or R Markdown files, so we have to pre-process those and commit the results to the repository. We decided that if we’re doing that, we might as well go the whole way, i.e., generate the HTML ourselves and commit that to the gh-pages branch rather than run Jekyll on the server at all.
  Another problem is that many things can only be expressed in Markdown by using HTML directly. In particular, there is no way to create div elements to represent things like callout boxes, exercises, lesson goals, and so on. We have resorted to using blockquotes for all of these, with some post-processing and CSS tricks to get the appearance we want.

Our next step (which we plan to implement in December 2015) is to take advantage of some of the extra features of one of the dialects of Markdown that Jekyll supports to solve the styling problem, so that we can store only the Markdown files in the GitHub repository, rather than the generated HTML. This will simplify things for newcomers, but we will still need custom build steps to handle Jupyter Notebooks, R Markdown, and other file formats, and the intermediate files produced by those build steps will still need to be kept in the repository.

Stepping back, what we have learned from wrangling formats is:

1. *There are no good answers.* Every currently-available option for publishing moderately complex material (such as lessons and scientific papers) is broken in some way.
2. *Fixing things is often a mistake.* Or rather, fixing things *frequently* is: as one of our instructors pointed out in the summer of 2015, every time he had taught a workshop in the previous three years, the process for setting up, formatting lessons, and so on had changed. We are now committed to updating our templates and processes no more than once a year.
3. *The best templates and platforms in the world won’t make writing lessons easy.* The best we can hope to achieve is to make it less hard.

## 8 TODO

We’ve learned a lot, and we’re doing a much better job of reaching and teaching people than we did three years ago, but there are still many things we need to improve.

### 8.1 Long-term assessment

Our biggest challenge is figuring out whether we are actually helping scientists get more science done, and if so, how, and how much. 5– 8 seem to show that we are, but we have not yet done a large-scale, long-term follow-up. This is partly because of a lack of resources, but it is also a genuinely hard problem: no one knows how to measure the productivity of programmers, or the productivity of scientists, and putting the two together doesn’t make the unknowns cancel out.

**Meeting our own standards**
One of the reasons we need to do long-term follow-up is to find out for our own benefit whether we’re teaching the right things the right way. As just one example, some of us believe that Subversion is significantly easier for novices to understand than Git because there are fewer places data can reside and fewer steps in its normal workflow. Others believe just as strongly that there is no difference, or that Git is actually easier to learn. While the large social network centered around GitHub is a factor in our choice as well, we would obviously be able to make better decisions if we had more quantitative data to base them on.

### 8.2 Too slow *and* too fast

Our second biggest challenge is the diversity of our learners’ backgrounds and skill levels. No matter what we teach, and how fast or how slow we go, 20% or more of the room will be lost, and there’s a good chance that a different 20% will be bored.

The obvious solution is to split people by level, but if we ask them how much they know about particular things, they regularly under- or over-estimate their knowledge. We have therefore developed a short pre-assessment questionnaire that asks them how easily they could do a small number of specific tasks. It is useful, in that it gives instructors some idea of who they’re going to be helping, but we have done nothing to validate the questions themselves, i.e., to ensure that respondents are interpreting them the same way that we are, or that their categorization of respondents corresponds in any meaningful way to actual proficiency. As mentioned in Section 8.1, we have been trying for several years to find the support needed to do rigorous assessment of this and other aspects of our program, but if funders are reluctant to invest in training, they are doubly reluctant to invest in measuring its effects.

### 8.3 “Is it supposed to hurt this much?”

Third, getting software installed is often harder than using it. This is a hard enough problem for experienced users, but almost by definition our audience is *inexperienced*, and our learners don’t (yet) know about system paths, environment variables, the half-dozen places configuration files can lurk on a modern system, and so on. Combine that with two versions of Mac OS X, three of Windows, and two oddball Linux distributions, and it’s almost inevitable that every time we introduce a new tool, it won’t work as expected (or at all) for at least one person in the room. Detailed documentation has not proven effective: some learners won’t read it (despite repeated prompting), and no matter how detailed it is, it will be incomprehensible to some, and lacking for others.

### 8.4 Editors

Editing text should be a minor problem, but if you’re standing in class telling three sets of users, "Now open Notepad++ if you’re on Windows, or Kate if you’re on Linux, or TextMate if you’re on a Mac, or whatever you want to use if you’re more advanced", and then demonstrate with whichever you have on your laptop (which looks different from what half of your learners are sitting in front of), you will cause mass confusion.

We therefore still use http://www.nano-editor.org/ as an editor in class, even though none of our instructors use it for real work. Arguments over this are another example of the bikeshedding discussed in Section 7: many people who are passionate about programming are also passionate (some might say “zealous”) about their favorite editor, and will argue about the relative merits of various choices at length.

The choice of editor is also an example of *expert blind spot*. People who know a subject well often have trouble re-imagining it through novice eyes, and hence underestimate how difficult “simple” tasks actually are for newcomers. For example, every reasonably experienced user of the shell knows that an editor can run inside a terminal window, so that a single fixture on the screen can play multiple roles. This is *not* obvious to newcomers, who are frequently confused when instructors move back and forth between an editor and a regular shell prompt in a single window.

### 8.5 Testing

We believe that software testing is important, but no longer include it in our core curriculum. The reason is that while it’s easy to teach the mechanics of using a unit testing framework ( https://en.wikipedia.org/wiki/XUnit), we do not know what tests to tell learners from dozens of different disciplines to write for their (very diverse) programs. In addition, while most research communities have a collective notion of what is “close enough” for laboratory work (“Physicists worry about decimal places, astronomers worry about the exponent, and economists are happy if they’ve got the sign right.”), similar heuristics have not yet emerged for key aspects of computational work. An attempt in 2014–15 to collect examples of actual tests from different domains didn’t achieve critical mass, but we hope to take another run at doing this.

### 8.6 Watching vs. doing

We try to make our teaching as interactive as possible, but we still don’t give learners hands-on exercises as frequently as we should. We also don’t give them as diverse a range of exercises as we should. This is simply due to a lack of time: two eight-hour days are as much as learners’ brains can handle, but not nearly enough to give them all the practice they need.

There is also a constant tension between having students do realistic exercises drawn from actual scientific work-flows, and giving them tasks that are small and decoupled, so that failures are less likely and don’t have knock-on effects when they occur. This is exacerbated by the diversity of learners in the typical workshop.

### 8.7 Less of a problem

One issue which is less of a problem than it used to be is financial sustainability. The “host site covers costs” model scales naturally with the number of workshops, while a growing number of organizations are keen to partner with us, primarily to build local capacity to run more work-shops when and as needed. While we do not wish to tempt fate, the Software Carpentry Foundation does seem to be headed toward financial stability.

## 9 Conclusions

To paraphrase William Gibson, the future is already here: it’s just that the skills needed to implement it aren’t evenly distributed. A small number of scientists can easily build an application that scours the web for recently-published data, launch a cloud computing node to compare it to home-grown data sets, and push the result to a GitHub account; others are still struggling to free their data from Excel and figure out which of the nine backup versions of their paper is the one they sent for publication.

The fact is, it’s hard for scientists to do the cool things their colleagues are excited about without basic computing skills, and impossible for them to know what other new things are possible. Our ambition is to change that: not just to make scientists more productive today, but to allow them to be part of the changes that are transforming science in front of our eyes. If you would like to help, we’d like to hear from you: please mail us at admin@software-carpentry.org.

## 10 Data availability

F1000Research: Dataset 1. Cumulative Number of Workshops over Time, 10.5256/f1000research.3536.d111654 ^26^

F1000Research: Dataset 2. Cumulative Number of Workshop Attendees over Time, 10.5256/f1000research.3536.d111655 ^27^

F1000Research: Dataset 3. Cumulative Number of Qualified Instructors over Time, 10.5256/f1000research.3536.d111656 ^28^
