# Supplementary material for: Software Carpentry: lessons learned
Source: F1000Res. 2016 Jan 28;3:62. Originally published 2014 Feb 19. [Version 2] doi: 10.12688/f1000research.3-62.v2 (PMC3976103; doi:10.12688/f1000research.3-62.v2)
Supplement: Peer review history

# Peer review: Referee response for version 1

**DOI:** 10.5256/f1000research.3787.r3816

**Published:** 2014-03-24

**Authors:** Hilmar Lapp

**Affiliations:** 1 National Evolutionary Synthesis Center (NESCent), Durham, NC, USA

The article describes some of the origins, driving motivations and lessons learned over the more than 15 years of iterative improvements and reboots of Software Carpentry, a brand of (meanwhile) travelling workshops teaching fundamental best practices in software engineering to programming scientists.

Software Carpentry has received wide acclaim, and helps fill critical gaps in a time when creating and using computational tools is becoming indispensable to increasingly many scientific fields. As such, the topic is of broad interest without question. The text is well-written, and in most places well argued. My only two overarching critiques are (1) that the author in some places seems to conflate cause and effect; and (2) that in some places I feel the reader is left hanging with a too little information. However, none of these rise to the level of calling into question the validity of the overall conclusions, and thus don't exceed what one might call "minor revisions".

Since this is an open review, I have chosen to record my detailed comments as public text annotations, using the Hypothes.is (http://hypothes.is) platform, with a transcription also provided below. A PDF version of the comments is also available.

 The Hypothes.is version of these comments can be accessed at this URL:

https://hypothes.is/stream#?user=hlapp&uri=http:%2F%2Ff1000research.com%2Farticles%2F3-62%2Fv1

Unfortunately, the ordering of the comments on Hypothes.is appears to be in reverse chronological order (most recent first), and the comments should therefore be read last to first to align with reading the text start to end.

Any comments or replies to these comments should be made using the *F1000Research *‘Add yours’ option but could also be added to Hypothes.is directly if desired.  

**Introduction**

1. Paragraph 1: *“hardware and algorithms are only two sides of the iron triangle of programming”*
  Is there a reference for this form of the Iron Triangle? Googling the phrase only turns up the well-known project management Iron Triangle, and its adaptation to software projects. The latter has Resources, Scope, and Time at its corners, not hardware, algorithms, and programming.
2. Paragraph 1: “ *desktop majority”*
  Do you mean the complement to those doing HPC? The phrase strikes me as needlessly cryptic. And are you sure that scientists developing HPC software are exempt from the trend you describe?
3. Paragraph 2: *“rarely if ever shown how to design a maintainable program in a systematic way”*
  Are they not in fact taught, even if only indirectly, that programs are typically not revisited again once passed on (to the course instructor, for example), and hence thinking about maintainability is wasted effort?
4. Paragraph 3: *“learning, and applying”*
  Assuming that learning binds to at least some of what we taught as well, the comma is extraneous. Or add a comma after “and applying”.
5. Paragraph 4: *“many researchers still find it hard to apply what we teach”*
  Researchers at-large, or researchers who participated in a Software Carpentry workshop?

**From red to green**

Version 1: Red light

 

1. Paragraph 1: *“i.e., to parallelize complex programs”*
  This seems more an example to me than a restatement of run before they could walk. Thus, this should be “e.g.” (or spelled out “for example”).
2. Paragraph 2: *“(then director of the Advanced Computing Laboratory at Los Alamos National Laboratory)”*
  Change parentheses to comma. The parenthetical phrase is important to make sense of the sentence. (And if similar contextual information can be given about Brent Gorda, i.e., information that helps to understand why he was invited, I suggest that be added too, as his current affiliation fails to explain that.)
3. Paragraph 2: *“In response, John Reynders (then director of the Advanced Computing Laboratory at Los Alamos National Laboratory) invited the author and Brent Gorda (now at Intel) to teach a week-long course on these topics to LANL staff. The course ran for the first time in July 1998, and was repeated nine times over the next four years.”*
  I suggest that the author highlights the major ways in which these courses differ from the SwC courses run today. As written now, deducing that from the two lessons learned is left as an exercise to the reader, and only those already familiar with SwC will know that indeed today's SwC workshops do differ in these ways.

Versions 2 and 3: Another red light

 

1. Paragraph 2: *“(even though a significant minority of their students, particularly those coming from non-CS backgrounds, have no more experience of practical software development than the average physicist”*
  Remove parentheses.
2. Paragraph 2: *“In the absence of an institutional mechanism to offer credit courses at some inter-departmental level, this course, like many other interdisciplinary courses, fell between two stools.”*
  Perhaps this would be beyond the scope of the paper as a commentary, but it would be interesting to see whether this is then different at decidedly interdisciplinary programs, for example programs interfacing computational biology / computer science / math.
3. Paragraph 3: *“It works too well to be interesting”*
  Based on context, “it” would be the SwC workshop or material. I suggest to reword so it is clear that it actually refers to the practices and tools being taught by SwC.
4. Paragraph 3: *“As long as universities reward research first, and supply teaching last, it is simply not in most computer scientists own best interests to offer this kind of course.”*
  If this is the main driver behind this kind of course not finding interest at university CS programs, is the situation then different at teaching-focused schools, such as small liberal arts colleges? There are small liberal arts colleges with strong CS programs; have they indeed been more welcoming to adopting SwC into their curricula?
5. Paragraph 4: *“This is partly because educators’ preferred file formats (Word, PowerPoint, and PDF) can’t be handled gracefully by existing version control systems, but more importantly, there simply isn’t a “culture of contribution” in education for projects like Software Carpentry to build on”*
  I'm not convinced that one isn't mostly or entirely a consequence of the other. Open source and collaborative development also was far less widespread in scientific software development before many of the barriers to that were significantly reduced by distributed version control such as Git, and usability and social coding focused resources such as Github. If the tools and file formats that are most widely used are simply refractory to collaboration, it's not a surprise if then a culture of collaboration is rare.
6. Paragraph 7: *“The sweet spot for this kind of training is therefore the first two or three years of graduate school. At that point, students have time (at least, more time than they’ll have once they’re faculty) and real problems of their own that they want to solve.”*
  Perhaps it's primarily the “real problems of their own” that provide the motivation for having the time (to learn about addressing them). I.e., percentage-wise, how many students does SwC get today who take the course primarily because they have time, and who do not yet have real problems of their own for which they hope to learn solutions?
  More importantly perhaps, does this not also point out a path for justifying the inclusion of SwC-inspired teaching units into undergraduate CS curricula? While for some (or most?) academic research career paths the relevance of version control mastery is perhaps less obvious, it's a qualification nearly all of industry ask of CS graduates applying for a software engineer position.

Version 4: orange light

 

1. Paragraph 1: *“The author rebooted Software Carpentry in May 2010 with support from Indiana University, Michigan State University, Microsoft, MITACS, Queen Mary University of London, Scimatic, SciNet, SHARCNet, and the UK Met Office.”*
  The backstory to what motivated (or necessitated?) the large consortium of funders is missing here. However, given the last paragraph in this section, it seems there would be interesting aspects of it that would help make setting up the argument. Does the large consortium reflect primarily wide buy-in to SwC's utility, or primarily the difficulty of obtaining enough funding from any one institution or partner? The last paragraph suggests it's the latter, but it's not clear.
2. Paragraph 1: *“MOOC”*
  Spell out at first use.
3. Paragraph 2: *“Open access publishing, crowd sourcing, and dozens of other innovations had convinced scientists that knowing how to program was now as important to doing science as knowing how to do statistics.”*
  Is there evidence or references for the factors the author enumerates constituting the major driving causes? More specifically, the list is conspicuously missing the explosion of data that had swept, and has continued to sweep into almost every scientific discipline. Data richness is enormously powerful for science, yet wrestling insight from it at this scale invariably and pervasively requires computational processing. Maybe this is part of the “dozens of other innovations”, but I would still argue that the data deluge has constituted a primary rather than a marginal driver of this landscape change.
4. Paragraph 4: *“Most importantly, the MOOC format didn’t work”*
  I think it's worth to qualify this statement in respect to the goals. As the paragraph goes on, it could be said that In some definition the MOOC format has worked (for example, compared to retention and completion rates of other MOOCs); the failure that the author reports presumably means chiefly that the goals laid out for a SwC course weren't met by the MOOC format.
5. Paragraph 5: *“The biggest take-away from this round was the need come up with a scalable, sustainable model. One instructor simply can’t reach enough people, and cobbling together funding from half a dozen different sources every twelve to eighteen months is a high-risk approach.”*
  For readers who aren't already fully on board with this, It would help to better set up the argument. Why is scaling up the model desirable or necessary? What is enough people? Couldn't funding also come from a single or few sources? Many courses are sustained by student tuition; how would this likely not work for SwC?

Version 5: green light

 

1. Paragraph 1 *“and backing from the Mozilla Foundation*”
  The difference in wording suggests that the Mozilla Foundation's backing didn't come in the form of a grant. Can it be spelled out (at least broadly) what that support consisted of?
2. Paragraph 1: *“This time, the model was two-day intensive workshops”*
  I'm curious as to why 2 days. The lessons learned stated earlier seem to say that attention drops after 3 days, not 2 days. Why was the decision made to shorten to 2 days, not 3 days?
3. Paragraph 1: *“The Hacker Within*”
  Is there no link or other reference available?
4. Paragraph 3: *“Switching to a “host site covers costs” model was equally important: funding is still needed for the coordinator positions (the author and two part-time administrative assistants at Mozilla, and part of one staff member’s time at the Software Sustainability Institute in the UK), but our other costs now take care of themselves.”*
  I'd find it really useful to spell this out a little more. What are “our other costs”? Instructor travel and expenses, room rental? What tasks do the coordinators perform, how does this scale? Or in other words, presumably there is a division between costs of operating that benefit from economies of scale, and those that do not. More insight into this division would be quite helpful as a lesson learned.
5. Paragraph 4: *“have grown steadily (Figure 1 and Figure 2).”*
  The figures suggest a tapering off in the recent past. Is this more likely a fluke due to limited or censored data, or is there a trend showing?
6. Figure 2 : *“Enrolment”  *
  Typo (one instead of two 'l')
7. Description of Figshare Data: *“Hopefully these two effects more or less cancel out and should not detract from the overall trend displayed.”*
  Hope is nice but not a good basis on which to base scientific conclusions. Do you have evidence that suggests that neither fraction of people is significant with respect to those enrolled and attending both days? Evidence that both fractions of people has stayed relatively constant over time, and not changed more recently?
8. Paragraph 5: *“80–90% of attendees typically report”*
  What does typically mean? 80-90% of all SwC enrolled students, or on average 80-90% of those enrolled in a workshop? I.e., how much variance is there between workshops?

 

**What we do**

 

1. Paragraph 5: *“While some people feel that using R instead of Python is like using feet and pounds instead of the metric system”*
  I have heard concerns and objections some people have with R's syntax and way of doing things. But every language (including Python) has its detractors, and I don't think the particular concerns with R are necessarily widely known let alone understood. So I would suggest to either delete this clause (is it really needed for the argument?), or if chosen to be left in place, to substantiate it, at least by giving a reference to a fuller discussion of R's problems.
2. Paragraph 5: *“now that we have enough instructors to be able to specialize”*
  It's probably not just a question of having instructors, but also of having demand for (and thus acceptance of) the SwC curriculum as useful in increasingly many disciplinary areas.
3. Paragraph 6: *“with the best”*
  Insert "even" before “with”.
4. Paragraph 7: “As this is usually several times more than a small registration fee would bring in, we usually choose the higher no-show rate as the lesser evil.”
  The biggest problem of a significant rate of no-shows is probably the fact that due to the space limitations other students who would have and benefitted from the course had to be denied because of the no-shows taking the space away. Have other possibilities to deter no-shows been explored (and if so, how effective have they been found)?
  If the no-show rate is somewhat predictable (and it sounds like it is), then wait-listed students could be told to show up anyway on the day of the course, because there would likely be enough no-shows to make room for them. Has this been tried, and to what extent does it work?
5. Paragraph 9: *“What does require permission is use of our name and logo, both of which are trademarked. We are happy to give such permission if we have certified the instructor and have a chance to double-check the content, but we do want a chance to check: we have had instances of people calling something “Software Carpentry” when it had nothing to do with what we usually teach. We’ve worked hard to create material that actually helps scientists, and to build some name recognition around it, and we’d like to make sure our name continues to mean something.”*
  This whole paragraph doesn't mention the words "brand", "brand recognition", and "brand reputation"; yet it is essentially about those concepts, isn't it? Why not say it directly?

**Small things add up**

Use what we teach

 

1. Paragraph 1: *“The (considerable) downside is that it can be quite difficult for newcomers to contribute material; we are therefore working to streamline that process.”*
  This needs some qualification to fully make sense as following from the preceding sentence. If the tools and approaches SwC teaches are good ones that "work", and SwC uses those tools and approaches itself, how can this be a downside, presuming that those able to contribute material are in fact familiar with those tools and approaches. I can imagine some ways in which this can still be a downside, but for clarity this should be spelled out better.

Keep experimenting

 

1. Paragraph 3: *“DiRAC”*
  Spell out. Also, how about a URL?
2. Paragraph 5: *“Many of our instructors also teach regular university courses, and several of them are now using part or all of our material as the first few lectures in them.”*
  Isn't this somewhat contradicting some lessons learned stated earlier, which seemed to say that for several reasons the SwC curriculum faces impossibly high barriers for integration into university curricula, at least in the current environment. If contrary to expectation this has now become possible, can something be learned from the cases where it has been successfully integrated?

**TODO**

Long-term assessment

 

1. Paragraph 1: *“no one knows how to measure the productivity of programmers, or the productivity of scientists”*
  I think this assertion needs better qualification to be really justified. Obviously, several ways to assess programmer productivity, and also scientist productivity, exist. Hiring and tenure committees regularly assess productivity of scientists. Arguably, the ways this is usually done suffers from various problems such as failing to encompass the full spectrum of products resulting from a scientist's work. Perhaps the author means that it is some of these shortcomings of current productivity assessment methods that effectively prevent measuring the productivity impact of SwC's teachings, but that needs to be spelled out better.

“Is it supposed to hurt this much?”

 

1. Paragraph 2: *“naive” *
  Is this meant to be "native"?

Teaching on the web

 

1. Paragraph 1: *“The fact that this is also true of most high-profile MOOCs is little comfort.”*
  If your goal is a high rate of retention and completion, that is. However, widening reach could also be a worthwhile goal. If a single MOOC reaches 10,000 students instead of 800 students reached by 20 physical SwC workshops, even a completion rate of only 10% will still have taught more students with the single MOOC than with the 20 physical workshops. MOOCs clearly aren't a panacea, and they may indeed be ill-suited to the learning objectives of SwC, but that and why this is so needs a little more depth to be convincing.

What vs. how

1. Paragraph 2: *“don’t as good a job”*
  Insert "do" after “don't”.
2. Paragraph 2: *“xUnit-style framework”*
  I'm embarrassed to ask what's an xUnit style framework. Spell out what that is, and/or add a reference or URL?

Standardization vs. customization

 

1. Paragraph 1: *“However, we do need to be more systematic about varying our content on purpose rather than by accident.”*
  As a reader, I feel left hanging by the section ending with this statement. Are there ideas about how this could be done, and to begin with, what were some of the problems encountered with the less systematic approach being practiced now? (The preceding text seems to only cite advantages.)

Watching vs. doing

 

1. Paragraph 1: *“We also don’t give them as diverse a range of exercises as we should, and those that we do give are often at the wrong level.”*
   How do you know that this is the case? From feedback alone, or are there other kinds of observations or evidence?
2. Paragraph 2: *“though we hope that will diminish as we organize and recruit along disciplinary lines instead of geographically”*
  Aren't you arguing above that diversity of backgrounds and starting skills is a constant challenge? It didn't seem from earlier arguments in the text that simply recruiting along a uniform discipline will address this problem.

Better teaching practices

 

1. Paragraph 1: *“We do our best to cover these ideas in our instructor training program, but are less good about actually applying them in our workshops.”*
  Is there some insight available into why instructors find it difficult to apply what they have been taught? Is it the imparting of these ideas that needs improvement, or are the ideas not as applicable in SwC as they were thought to be, or is there simply heterogeneity in that some ideas are much easier to apply than others? If the latter, which ones fall into which category?

 

**Conclusions**

 

1. Paragraph 1: *“To paraphrase William Gibson”*
  I notice that it's not clear to what exact piece or event to source this. Perhaps still link to the William Gibson Wikiquote page, which includes the quote and its provenance?

I have read this submission. I believe that I have an appropriate level of expertise to confirm that it is of an acceptable scientific standard.

# Peer review: Referee response for version 1

**DOI:** 10.5256/f1000research.3787.r3815

**Published:** 2014-02-26

**Authors:** Philip Guo

**Affiliations:** 1 Department of Computer Science, University of Rochester, Rochester, NY, USA

This article is a retrospective on the past 15 years of the author leading the Software Carpentry effort to educate scientists about the practical value of computational tools. It also describes a set of instructional practices that have worked well (and some that have not worked so well) in this setting. It concludes with ongoing and future work to scale up Software Carpentry in light of large variations in instructor and student backgrounds, and continual changes in modern computational tools.

This article is a great fit for F1000 due to the topic's relevance to researchers in life sciences (and across a diverse array of science fields), and to the author's cogent firsthand accounts of his experiences and reflections on a subject matter which he is well-suited to discuss.

My main high-level comment is that the language is colloquial in many parts of this article, with lots of asides enclosed (in parentheses). That is probably fine for an opinion-based article, and it makes the writing more personal and approachable. But the author should be aware that this is how the article appears to a first-time reader.

Here are some more detailed comments, none of which are pressing:

- "From red to green" -- it took me a while to understand the "red", "orange", "green" light analogy the author was making in this section. That seems to be culturally specific. (I don't think I've seen an orange traffic light.)
- "Versions 2 and 3: Another red light" - I didn't understand why these were two separate versions. Maybe it's simpler just to call this Version 2 and update the subsequent version numbers?
- "It works too well to be interesting" -- This blurb felt a bit harsh toward CS professors. It makes it sound like they teach only topics that lead to new publishable research. In my experience, teaching and research are fairly decoupled, so professors have no qualms about teaching materials from, say, 30-year-old compilers or databases textbooks, which are obviously not leading to new research. Perhaps a more likely explanation, which the author points out later in the article, is that there simply isn't room in CS curricula to offer these sorts of Software Carpentry-like materials, and nobody vouches strongly enough for them.
- Typo in caption: "Enrolment figures" -> "Enrollment figures"
- "What we do" - "Day 1 a.m.", etc. -- that's hard to parse. I thought the author meant "1am" like they were offering a class at 1 in the morning. Same with "1pm", "2am", "2pm". "Day 1 - morning" would be clearer.
- "during the practical" - I'm not familiar with this phrase. Is that a typo, or a figure of speech?
- "It's a refreshing change from teaching undergraduate calculus." -- would Software Carpentry instructors ordinarily teach calculus? Seems more like they would be teaching physics or programming or something.

I have read this submission. I believe that I have an appropriate level of expertise to confirm that it is of an acceptable scientific standard.

# Peer review: Referee response for version 1

**DOI:** 10.5256/f1000research.3787.r3814

**Published:** 2014-02-24

**Authors:** Juha Sorva

**Affiliations:** 1 Department of Computer Science, Aalto University, Espoo, Finland

This is an insightful and well-written commentary on a timely topic. The article documents the past and present practices of Software Carpentry - a project for teaching scientists about computing - and reflects on the project's successes and failures. In doing so, it provides concrete examples of the teaching practices used as well as those discarded. Moreover, the article helps the reader to understand how teaching scientists to about computing is different from teaching computer science majors - a matter that is central to the efforts of Software Carpentry and to the interests of the growing numbers of scientists who need computing skill to work efficiently.

The commentary is well grounded in evidence from the research literature as well as the author's lengthy experience with the project. The achievements and challenges of Software Carpentry are discussed realistically and critically.

**Additional comment**: There is only so much you can learn in two days (the length of Software Carpentry's current workshops), and whatever you learn in that time is unlikely by itself to change your research practices dramatically. What would be interesting to know in the future is whether and how the workshop participants go about building their computing skills after attending a workshop.

I have read this submission. I believe that I have an appropriate level of expertise to confirm that it is of an acceptable scientific standard.
